# Study on the correlation between bioelectrical impedance analysis index and protein energy consumption in maintenance dialysis patients

**DOI:** 10.1186/s12937-023-00890-5

**Published:** 2023-11-09

**Authors:** Weina Wang, Xinxuan Meng, Jiaojiao Liu, Xiaowei Lou, Ping Zhang, Peipei He, Jianghua Chen, Jing Yuan

**Affiliations:** 1 Kidney Disease Center, First Affiliated Hospital, College of Medicine, Zhejiang University, Hangzhou, 310000 China; 2 College of Medicine, Zhejiang University, Hangzhou, 310000 China; 3 Hebei ophthalmology hospital, Xingtai, 054000 China

**Keywords:** Bioelectrical impedance analysis, Maintenance dialysis, Protein-energy wasting, Diagnostic model

## Abstract

**Background:**

Protein-energy wasting (PEW) has been reported to be pretty common in maintenance dialysis patients. However, the existing PEW diagnostic standard is limited in clinical use due to the complexity of it. Bioelectrical impedance analysis (BIA), as a non-invasive nutritional assessment method, can objectively and quantitatively analyze the changes of body tissue components under different nutritional states. We aim to explore the association between PEW and BIA and establish a reliable diagnostic model of PEW.

**Methods:**

We collected cross-sectional data of 609 maintenance dialysis patients at the First Affiliated Hospital, College of Medicine, Zhejiang University. PEW was diagnosed according to International Society of Renal Nutrition and Metabolism (ISRNM) criteria. Among them, 448 consecutive patients were included in the training set for the establishment of a diagnostic nomogram. 161 consecutive patients were included for internal validation. 52 patients from Zhejiang Hospital were included for external validation of the diagnostic model. Correlation analysis of BIA indexes with other nutritional indicators was performed. Logistic regression was used to examine the association of BIA indexes with PEW. 12 diagnostic models of PEW in maintenance dialysis patients were developed and the performance of them in terms of discrimination and calibration was evaluated using C statistics and Hosmer–Lemeshow-type χ2 statistics. After comparing to existing diagnostic models, and performing both internal and external validation, we finally established a simple but reliable PEW diagnostic model which may have great value of clinical application.

**Results:**

A total of 609 individuals from First Affiliated Hospital, College of Medicine, Zhejiang University and 52 individuals from Zhejiang Hospital were included. After full adjustment, age, peritoneal dialysis (compared to hemodialysis), subjective global assessment (SGA, compared to non-SGA) and water ratio were independent risk factors, while triglyceride, urea nitrogen, calcium, ferritin, BCM, VFA and phase angle were independent protective factors of PEW. The model incorporated water ratio, VFA, BCM, phase angle and cholesterol revealed best performance. A nomogram was developed according to the results of model performance. The model achieved high C-indexes of 0.843 in the training set, 0.841 and 0.829 in the internal and external validation sets, respectively, and had a well-fitted calibration curve. The net reclassification improvement (NRI) showed 8%, 13%, 2%, 38%, 36% improvement of diagnostic accuracy of our model compared with “PEW score model”, “modified PEW score model”, “3-index model”, “SGA model” and “BIA decision tree model”, respectively.

**Conclusions:**

BIA can be used as an auxiliary tool to evaluate PEW risk and may have certain clinical application value.

**Supplementary Information:**

The online version contains supplementary material available at 10.1186/s12937-023-00890-5.

## Introduction

Protein-energy wasting (PEW) is a common complication in maintenance dialysis patients. The International Society of Renal Nutrition and Metabolism (ISRNM) described PEW as a group of clinical syndromes such as muscle tissue wasting and malnutrition in chronic kidney disease (CKD) patients but not just a state of malnutrition [1]. In 2008, ISRNM members proposed the diagnostic criteria of PEW, covering laboratory test results, anthropometric indexes and dietary intake [1]. However, diagnostic standards have been controversial since then. In clinical practice, it’s difficult to assess PEW due to complex diagnostic criteria, resulting in insufficient nutritional support or excessive nutritional intervention [2–5]. There are some specific scores, such as the subjective global assessment (SGA) and malnutrition-inflammation score (MIS) which show prognostic value in patients on hemodialysis [6, 7]. However, the SGA method has defects in accuracy and objectivity [8, 9]. And whether MIS is suitable for Chinese dialysis population still needs to be confirmed through large-scale clinical research though it has been proven to have high validity of clinical application [10]. Bioelectrical impedance analysis (BIA), as a body composition analysis technique, uses body composition impedance to calculate body composition indicators such as muscle, fat, cell mass and volume load status. It can quantitatively analyze various body tissue components, such as skeletal muscle mass (SMM), soft lean mass (SLM), visceral fat area (VFA), fat free mass (FFM), body fat percentage (BFP), total body cell mass (BCM), extracellular water (ECW), intracellular water (ICW), as well as direct measures like impedance and phase angle. Several studies have confirmed that there’s a correlation between BIA and nutritional indicators, and BIA indexes may be good predictors of PEW [11–15]. Effective model for BIA to evaluate PEW risk has not been established, and whether BIA indexes can be used as diagnostic factors remains controversial. Therefore, we aimed to explore the diagnostic value of BIA indexes for PEW.

## Materials and methods

### Study participants

In this study, we used convenience sampling method to select 609 patients in the First Affiliated Hospital, Zhejiang University School of Medicine from January 2019 to June 2019 and 52 patients in Zhejiang Hospital from October 2018 to September 2020 as study participants. The inclusion criteria were: (1) patients aged 18–80 years (2) patients diagnosed as end-stage renal disease (ESRD) receiving renal replacement therapy (3) patients receiving regular peritoneal dialysis or hemodialysis for more than 3 months. The exclusion criteria were: (1) patients in unstable health status (peritoneal dialysis patients with peritonitis within three months, combined with acute or chronic infection, heart failure, active liver disease, malignant tumor, acute cardiovascular and cerebrovascular disease, tuberculosis, peptic ulcer and other diseases) (2) peritoneal dialysis and hemodialysis were performed at the same time (3) patients treated with glucocorticoids or other immunosuppressant (4) patients with metal stents or amputation (5) patients with mental illness. The study was approved by the local ethics committees and conducted in accordance with the principles of the Declaration of Helsinki.

### Bioelectrical impedance measuring method

We used Korea InBody S10 Biospace multi-frequency bioelectrical impedance body composition analyzers, which apply the principle of bioelectrical impedance spectrum, and accurately calculate body composition through current measurement in different frequency ranging from 5 to 1000 kHz. The measurement time point was within 15 min after the end of dialysis. All BIA indexes were obtained using foot to hand technology.

### Data collection

The data we collected were as follows: (1) Clinical data, including age, sex and dialysis duration; (2) Pre-dialysis laboratory test results, including albumin, prealbumin, urea clearance index (Kt/V), cholesterol, hemoglobin, serum creatinine, urea nitrogen, phosphorus, calcium, parathyroid hormone, serum iron, ferritin, C-reactive protein and normalized protein catabolic rate (nPCR); (3) Post-dialysis anthropometric indexes, including arm circumference, arm muscular circumference (AMC), triceps skinfold thickness (TST) and body mass index (BMI); (4) BIA indexes, including FFM, FAT, BCM, BFP, SLM, ECW, water ratio, VFA, impedance and phase angle. Among them, impedance and phase angle were measured at 5 kHz and all BIA indexes were performed using the whole body measurement method. (5) SGA questionnaire (Table supplementary 1). Kt/V=-In(R-0.008t)+(4-3.5R) × UF/W, (where R is the ratio of urea nitrogen after dialysis and urea nitrogen before dialysis; t is dialysis duration for one time; ln is the natural logarithm; UF is the ultrafiltration volume; W is the patient’s body weight after dialysis) [16]. nPCR = urea nitrogen before penetration / [25.8 + 1.15] × spKt/V + 56.4/spKt/V] + 0.168 [17]. AMC = upper arm circumference − 0.314 × triceps skinfold thickness [18]. BMI = weight / height^2^ [19]. Water ratio = extracellular water (ECW)/total body water (TBW). Body surface area (BSA, cm^2^) = 0.0003207*weight^0.7285−0.0188*log(weight)^*height^0.3^ [20, 21]. Overhydration (OH) = 1.14*ECW-0.43*ICW-0.11*Weight. Relative OH = OH/ECW. Lean tissue index (LTI) = SLM/height^2^. Fat tissue index (FTI) = FAT/height^2^ [22].

### Diagnostic standard of PEW

According to the diagnostic criteria of ISRNM [1], (1) laboratory test results: albumin < 38 g / L, prealbumin < 300 mg / L, cholesterol < 2.59 mmol / L; (2) body mass index: BMI < 23 kg / m^2^ or dry weight loss (3 months > 5% or 6 months > 10%); (3) muscle mass index: AMC decreased (3 months > 5% or 6 months > 10%); (4) dietary intake: nPCR < 0.8 g / (kg•d). PEW can be diagnosed only when a patient has at least three out of the above four groups of indicators, while at least one indicator meet the requirements in each group.

### Statistical analyses

**Fig. 1:**
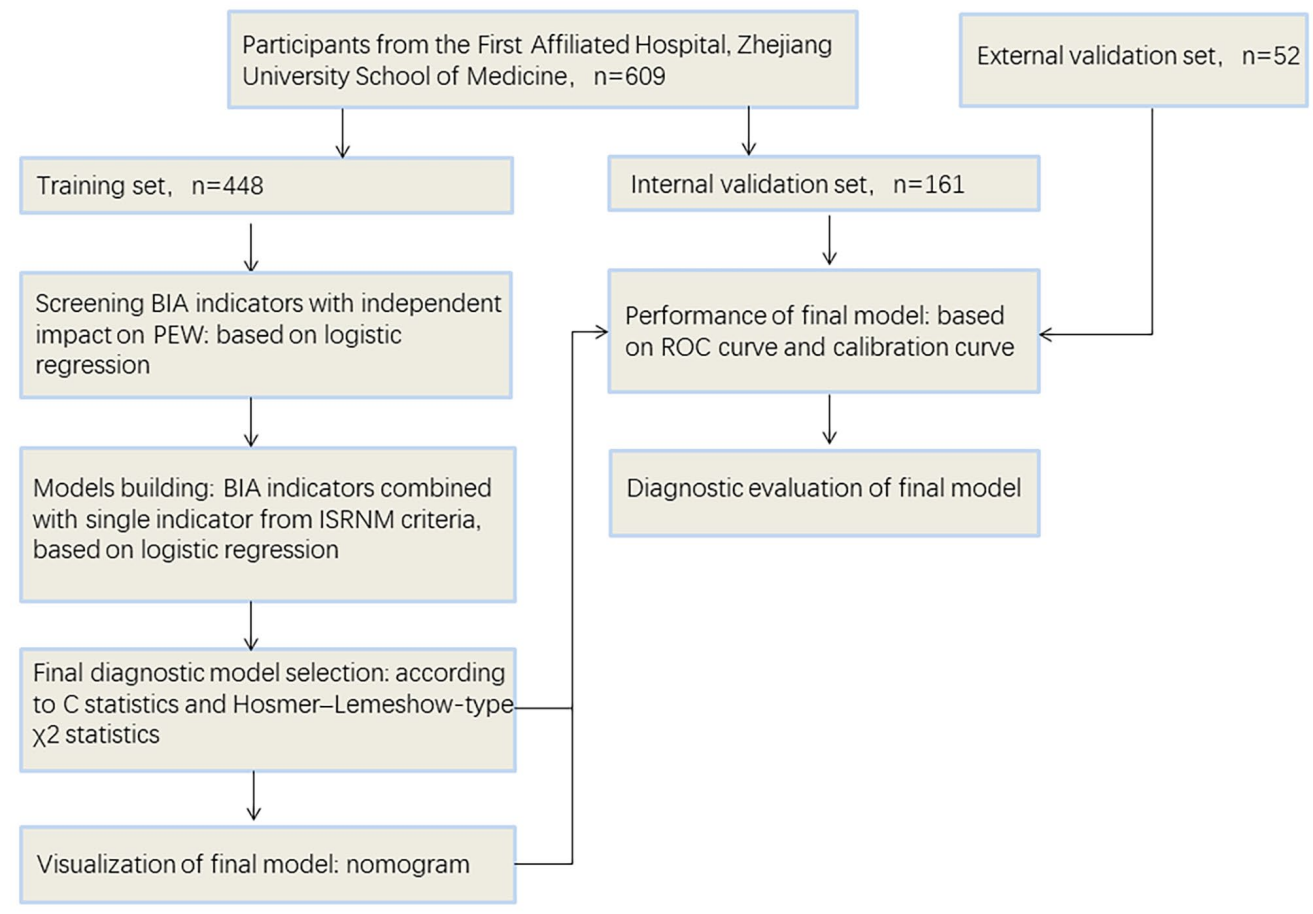
Analysis flowchart. Participants from First Affiliated Hospital, Zhejiang University School of Medicine were randomly divided into training set and validation set according to the ratio of 7:3. 52 patients from Zhejiang Hospital were used for external validation. The figure shows the data analysis conducted for each dataset. PEW, protein-energy wasting; BIA, bioelectrical impedance analysis; ISRNM, International Society of Renal Nutrition and Metabolism; ROC, receiver operating characteristic

Participants from internal set were randomly divided into training set and validation set according to the ratio of 7:3. Normality of distribution was tested with *Kolmogorov-Smirnov’s* test. Quantitative variables are presented as means ± standard deviations (SDs) and were compared using *t*-test for normally distributed data. Non-normally distributed variables are summarized as medians and interquartile ranges (IQRs), and were compared using *Mann–Whitney* test. Categorical variables are expressed as percentages or frequencies and were assessed with the *chi-squared* test. To determine the association of BIA indexes with nutritional indicators, a correlation heatmap was performed after using *Pearson’s* correlation analysis. Furthermore, logistic regression was used to examine the association between BIA indexes and PEW. After selecting BIA indexes that are independent influencing factors of PEW, 12 models were constructed to generate probability of PEW by using logistic regression. Variance inflation factors were used to test the collinearity among variables. Discrimination was quantified by calculating C statistics developed for models. *Hosmer–Lemeshow- type χ2* statistics were used to assess calibration. A nomogram was developed according to the results of model performance of data from training set. Its discriminatory ability was validated in internal and external validation sets by using receiver operating characteristic (ROC) curves and the calibration was assessed with calibration curves in internal and external validation sets for which bootstraps with 40 resamples were used for calculations. Diagnostic test evaluation was conducted to compare the performance of the new model with previous models (Fig. 1 for detailed analysis flowchart). Indexes included in the diagnostic test evaluation can be calculated as follows: Sensitivity = true positives/ (true positives + false negatives). Specificity = true negatives / (true negatives + false positives). Positive predictive value (PPV) = true test positives / all test positives. Negative predictive value (NPV) = true test negatives / all test negatives. Net reclassification index (NRI) = (p_up,events_ - p_down,events)_ - (p_up,nonevents_ - p_down,nonevents_) [23]. SPSS 25.0 (IBM, Armonk, NY, USA) and R statistical software version 4.1.2 (R Foundation) were used for the statistical analysis and P-values < 0.05 were considered statistically significant.

## Results

### Baseline characteristics

Participants from the First Affiliated Hospital, Zhejiang University School of Medicine were randomly divided into training set and validation set according to the ratio of 7:3. There’s no statistically significance of indexes between training set and validation set (Table supplementary 2). Table 1 shows the characteristics of participants from training set. At baseline, 109 participants (24.3%) were diagnosed as PEW according to diagnostic criteria of ISRNM [1]. Univariate analysis revealed that compared with those without PEW, participants with PEW were more likely to be older, have higher probability of malnutrition according to SGA, have lower albumin, prealbumin, cholesterol, triglyceride, nPCR, serum creatinine, hemoglobin, urea nitrogen, calcium, serum iron, ferritin, arm circumference, AMC, TST, BMI, BCM, SLM, VFA and phase angle, but higher water ratio, ECW, and C-reactive protein. (all above p < 0.05)

**Table 1:** Baseline characteristics of participants from training set

Characteristics	PEW of training set(n = 448)		P value
	No(n = 339,75.7%)	Yes(n = 109,24.3%)	
Sex			
Male	187(55.2%)	55(50.5%)	0.237
Female	152(44.8%)	54(49.5%)	
Age, y	53.49 ± 13.29	56.37 ± 14.16	0.014*
Dialysis modality			
Peritoneal dialysis	177(52.2%)	58(53.2%)	0.890
Hemodialysis	162(47.8%)	51(46.8%)	
Dialysis duration, m	36.5(17.8,64.3)	37.0(18.0,67.0)	0.977
SGA			
Yes	40(11.8%)	30(27.5%)	< 0.001**
No	299(88.2%)	79(72.5%)	
Albumin, g/L	40.77 ± 3.92	37.64 ± 4.07	< 0.001**
Prealbumin, g/L	40.30 ± 9.11	33.97 ± 0.46	< 0.001**
Cholesterol, mmol/L	4.29 ± 1.09	3.48 ± 1.02	< 0.001**
Triglyceride, mmol/L	1.86(1.26,2.66)	1.33(0.94,2.00)	< 0.001**
nPCR, g/(kg·d)	1.03 ± 0.32	0.84 ± 0.31	< 0.001**
C-reactive protein, mg/L	1.61(0.53,4.35)	1.97(0.61,7.33)	0.038*
Serum creatinine, µmol/L	929.67 ± 278.77	862.72 ± 278.23	0.005**
Kt/V	1.83 ± 0.48	1.79 ± 0.53	0.381
Hemoglobin, mg/L	106.87 ± 14.13	102.90 ± 15.72	0.002**
Urea nitrogen, mmol/L	23.62 ± 6.03	21.84 ± 6.51	0.001**
Calcium, mmol/L	2.27 ± 0.23	2.22 ± 0.21	0.022*
Phosphorus, mmol/L	1.80 ± 0.48	1.73 ± 0.53	0.072
Parathyroid hormone, pg/mL	283.3(154.0,451.0)	272.5(138.1,480.0)	0.398
Serum iron, µmol/L	12.1(9.4,15.3)	10.2 (7.7,14.2)	< 0.001**
Ferritin, pg/mL	165.1(73.3,364.6)	130.4(51.4,300.9)	0.007**
Arm circumference, cm	27.49 ± 4.23	25.23 ± 2.94	< 0.001**
AMC, cm	22.27 ± 2.71	20.71 ± 1.98	< 0.001**
TST, mm	16.24(12.74,19.75)	13.38(10.67,17.52)	< 0.001**
BMI, kg/m^2^	22.42 ± 3.33	19.87 ± 2.54	< 0.001**
FFM, kg	41.2(31.0,51.6)	39.7(27.1,51.1)	0.333
FAT, kg	18.45(11.05,31.95)	16.30(9.00,27.10)	0.068
BFP, %	28.77(18.22,47.67)	26.28(19.22,43.84)	0.739
BCM, kg	28.24 ± 7.17	26.23 ± 6.63	0.001**
Water ratio(ECW/TBW),%	38.73 ± 2.78	40.50 ± 4.96	< 0.001**
ECW, kg	12.87 ± 4.40	14.27 ± 7.69	0.019*
SLM, kg	41.40 ± 10.25	38.41 ± 9.52	0.001**
VFA, cm^2^	55.5(32.7,78.4)	47.4(27.9,66.8)	0.002**
Impedace, Ω	1005.64 ± 187.51	1030.94 ± 208.70	0.160
Phase angle, °	6.34 ± 2.17	5.47 ± 1.61	< 0.001**

SGA-Yes: malnutrition evaluated through subjective global assessment. SGA-No: good nutritional status evaluated through subjective global assessment. PEW, protein-energy wasting; nPCR, normalized protein catabolic rate; AMC, arm muscle circumference; TST, triceps skinfold thickness; BMI, body mass index; FFM, fat free mass; BFP, body fat percentage; BCM, body cell mass; ECW, extracellular water; SLM, soft lean mass; VFA, visceral fat area. *: p < 0.05; **: p < 0.01

### Association of BIA indexes with nutrition-related indicators

**Fig. 2:**
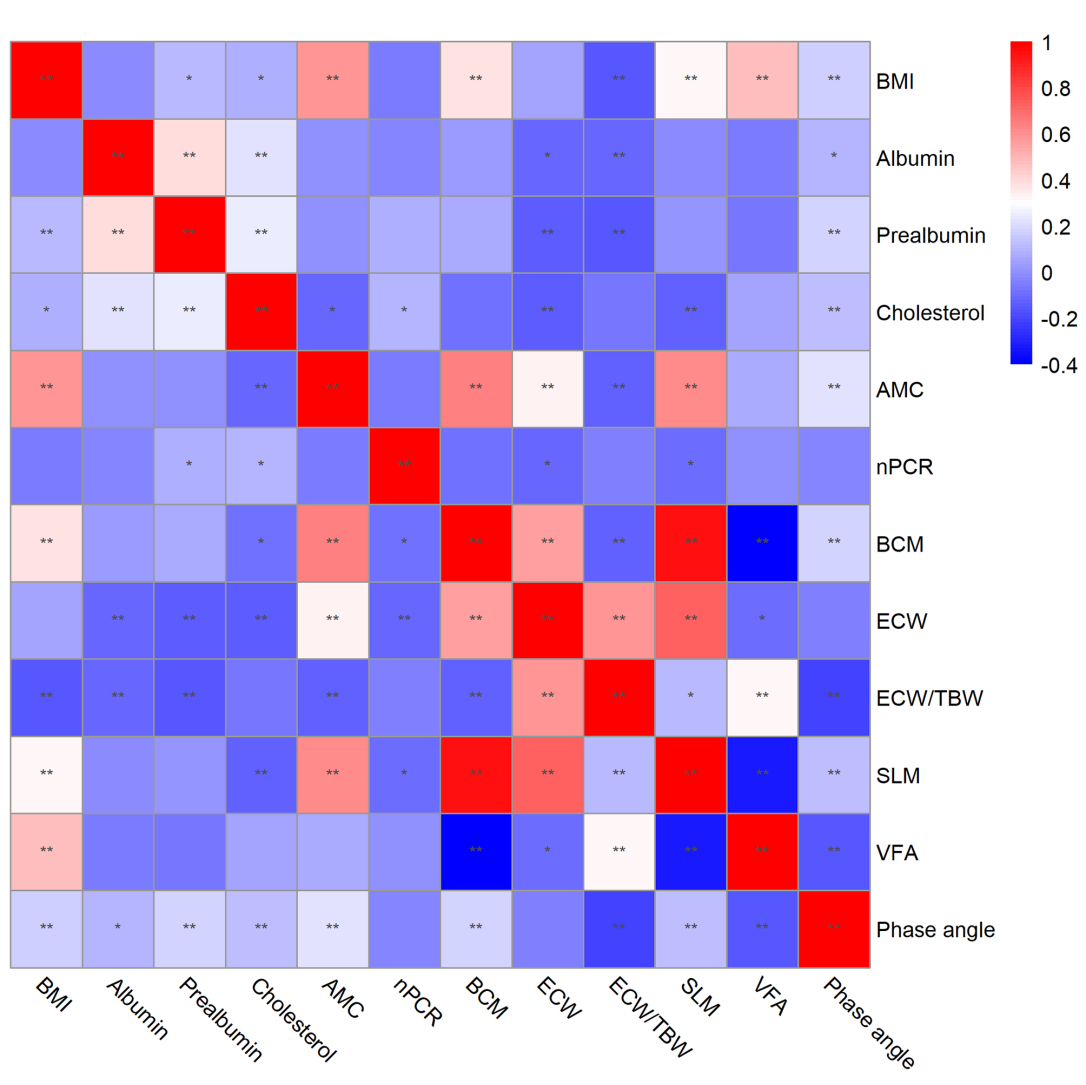
Correlation heatmap. The heatmap displays correlation of BIA indexes (BCM, ECW, water ratio, SLM, VFA, phase angle), nutritional indicators (nPCR), anthropometric indicators (BMI, AMC) and laboratory indicators (albumin, prealbumin, cholesterol). Warm color indicates a positive correlation between two indicators, while cool color indicates a negative correlation between two indicators. BMI, body mass index; AMC, arm muscle circumference; nPCR, normalized protein catabolic rate; BCM, body cell mass; ECW, extracellular water; TBW, total body water; SLM, soft lean mass; VFA, visceral fat area.*: P < 0.05; **: P < 0.01

Figure 2 shows the association among BIA indexes, nutritional indicators, anthropometric indicators and laboratory indicators. BCM was positively correlated with BMI and AMC. ECW was negatively correlated with albumin, prealbumin, cholesterol and nPCR. Water ratio was negatively correlated with BMI, albumin, prealbumin and AMC. SLM was negatively correlated with cholesterol and nPCR, and positively correlated with AMC. VFA was positively correlated with BMI. Phase angle was negatively correlated with BMI, albumin, prealbumin, cholesterol and AMC. (all above p < 0.05)

### Multivariate binary logistic regression analysis

**Fig. 3:**
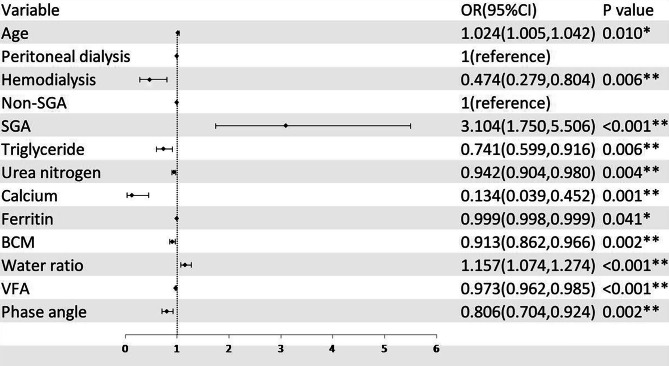
Forest plot. Logistic regression is applied to screen for independent influencing factors of PEW. OR > 1 indicates an independent risk factor, while OR < 1 indicates an independent protective factor for PEW. In this figure, dialysis modality(reference group: peritoneal dialysis) and SGA result(reference group: non-SGA) are categorical variables. SGA, malnutrition evaluated through subjective global assessment; BCM, body cell mass; VFA, visceral fat area; OR, odds ratio. *: P < 0.05; **: P < 0.01

After excluding indexes in the diagnostic criteria of PEW, then selecting the factors that were statistically significant in the results of univariate analysis, and empirically incorporating sex and dialysis modality variables, there were 20 variables, which may be influencing factors of PEW, including age, sex, dialysis modality, SGA, triglyceride, C-reactive protein, serum creatinine, hemoglobin, urea nitrogen, calcium, serum iron, ferritin, arm circumference, TST, BCM, water ratio, ECW, SLM, VFA, and phase angle. Further, the influencing factors of PEW were analyzed by stepwise backward multivariate binary logistic regression. The results showed that age (OR = 1.024, 95%CI: 1.005 ~ 1.042), SGA (compared to non-SGA, OR = 3.104, 95%CI: 1.750 ~ 5.506), water ratio (OR = 1.157, 95%CI: 1.074 ~ 1.274) were risk factors of PEW, while hemodialysis (compared to peritoneal dialysis, OR = 0.474, 95%CI: 0.279 ~ 0.804), triglyceride (OR = 0.741, 95%CI: 0.599 ~ 0.916), urea nitrogen (OR = 0.942, 95%CI: 0.904 ~ 0.980), calcium (OR = 0.134, 95%CI: 0.039 ~ 0.452), ferritin (OR = 0.999, 95%CI: 0.998 ~ 0.999), BCM (OR = 0.913, 95%CI: 0.862 ~ 0.966), VFA (OR = 0.973, 95%CI: 0.962 ~ 0.985) and phase angle (OR = 0.806, 95%CI: 0.704 ~ 0.924) were the protective factors of PEW (Fig. 3).

### Diagnostic models building

Models including single indicator from ISRNM criteria (BMI, albumin, prealbumin, cholesterol, AMC, nPCR) with (model 1-6b) or without (model 1-6a) 4 BIA indexes (water ratio, VFA, BCM, phase angle) were constructed respectively by the method of logistic regression. C statistics and H-L type χ2 statistics are shown in Table 2. Models from b group have higher C statistics than models from a group, indicating an additional prediction effect of BIA beyond single ISRNM indicators. Among the 12 models, model 4b (4 BIA indexes + cholesterol) has good performance in both discrimination and calibration. Result of collinearity diagnosis for model 4b is shown in Table supplementary 3, indicating no indicative serious collinearity. Through this diagnostic model, the PEW risk can be calculated by the following formula:

**Table 2:** Models including BIA indexes and ISRNM indicators

Models	Indexes in model	Discrimination		Calibration	
		C statistics	P value	H-L-type χ2	P value
Model 1a	BMI	0.742(0.703–0.780)	< 0.001**	27.597	0.001
Model 1b	BIA indexes + BMI	0.769(0.729–0.810)	< 0.001**	10.065	0.261
Model 2a	albumin	0.751(0.706–0.796)	< 0.001**	58.122	< 0.001
Model 2b	BIA indexes + albumin	0.818(0.779–0.858)	< 0.001**	5.194	0.737
Model 3a	prealbumin	0.676(0.625–0.727)	< 0.001**	32.637	< 0.001
Model 3b	BIA indexes + prealbumin	0.783(0.742–0.824)	< 0.001**	4.034	0.854
Model 4a	cholesterol	0.762(0.720–0.804)	< 0.001**	76.201	< 0.001
Model 4b	BIA indexes + cholesterol	0.843(0.806–0.880)	< 0.001**	6.197	0.625
Model 5a	AMC	0.685(0.642–0.728)	< 0.001**	6.763	0.562
Model 5b	BIA indexes + AMC	0.767(0.726–0.809)	< 0.001**	14.638	0.067
Model 6a	nPCR	0.724(0.678–0.771)	< 0.001**	45.852	< 0.001
Model 6b	BIA indexes + nPCR	0.844(0.806–0.881)	< 0.001**	25.438	0.001

BIA indexes: Water ratio, VFA, BCM and phase angle. ISRNM indicators: albumin, prealbumin, cholesterol, AMC and nPCR. Model a: models including one single ISRNM indicator. Model b: models including BIA indexes and one single ISRNM indicator. BIA, bioelectrical impedance analysis; ISRNM, International Society of Renal Nutrition and Metabolism; BMI, body mass index; AMC, arm muscle circumference; nPCR, normalized protein catabolic rate. *: p < 0.05; **: p < 0.01

$$\begin{array}{l}p\, = \,1/[1\, + \,exp\,( - \,3.779\, + \,1.105\,*cholesterol\, - \,0.142\,*water\,\\ratio\, + \,0.188\,*\,phase\,angle\, + \,0.030\,*\,VFA\, + \,0.133\,*\,BCM)]\end{array}$$

### Diagnostic nomogram

**Fig. 4:**
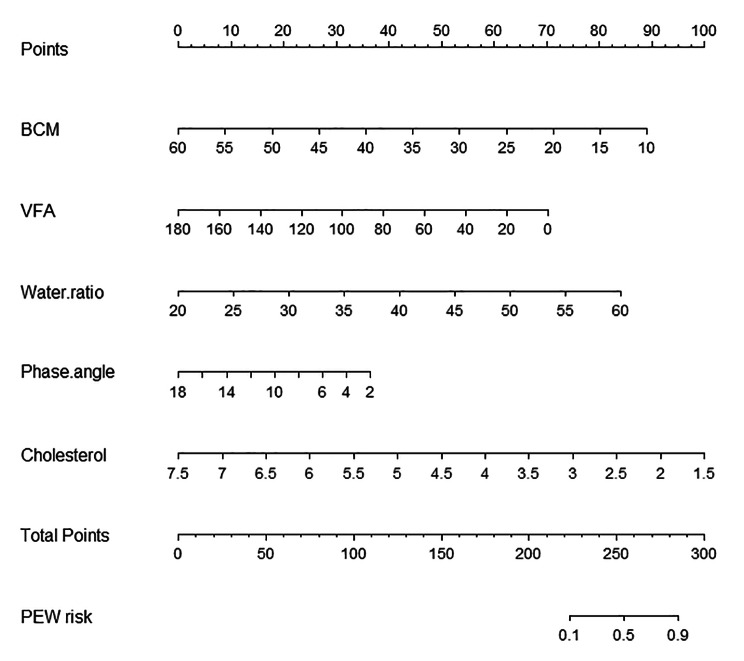
The diagnostic nomogram of PEW in maintenance dialysis patients based on the training set. The value of each variable was scored on a point scale from 0 to 100, after which the scores for each variable were added together. That sum is located on the total points axis, which enables us to predict the PEW risk. PEW positive diagnosis is defined as PEW risk > 0.29. If indexes of one maintenance dialysis patient are as follows: BCM 15 kg (scoring 80 on points axis), VFA 20 cm2 (scoring 62 on points axis), water ratio 40% (scoring 42 on points axis), phase angle 5°(scoring 30 on points axis), cholesterol 6 mmol/L (scoring 25 on points axis), the total score can be calculated as 239 on total points axis, with corresponding PEW risk 33% according to PEW risk axis. BCM, body cell mass; VFA, visceral fat area; PEW, protein-energy wasting

To visualize the final diagnostic model, a nomogram was constructed (Fig. 4). To calculate a patient’s PEW probability, points for each parameter are assigned by corresponding values from the “points” axis, and sum of the points is plotted on “total points” axis. The patient’s PEW probability is the value at a vertical line from corresponding total points.

### Performance of the diagnostic model in internal and external validation sets

**Fig. 5:**
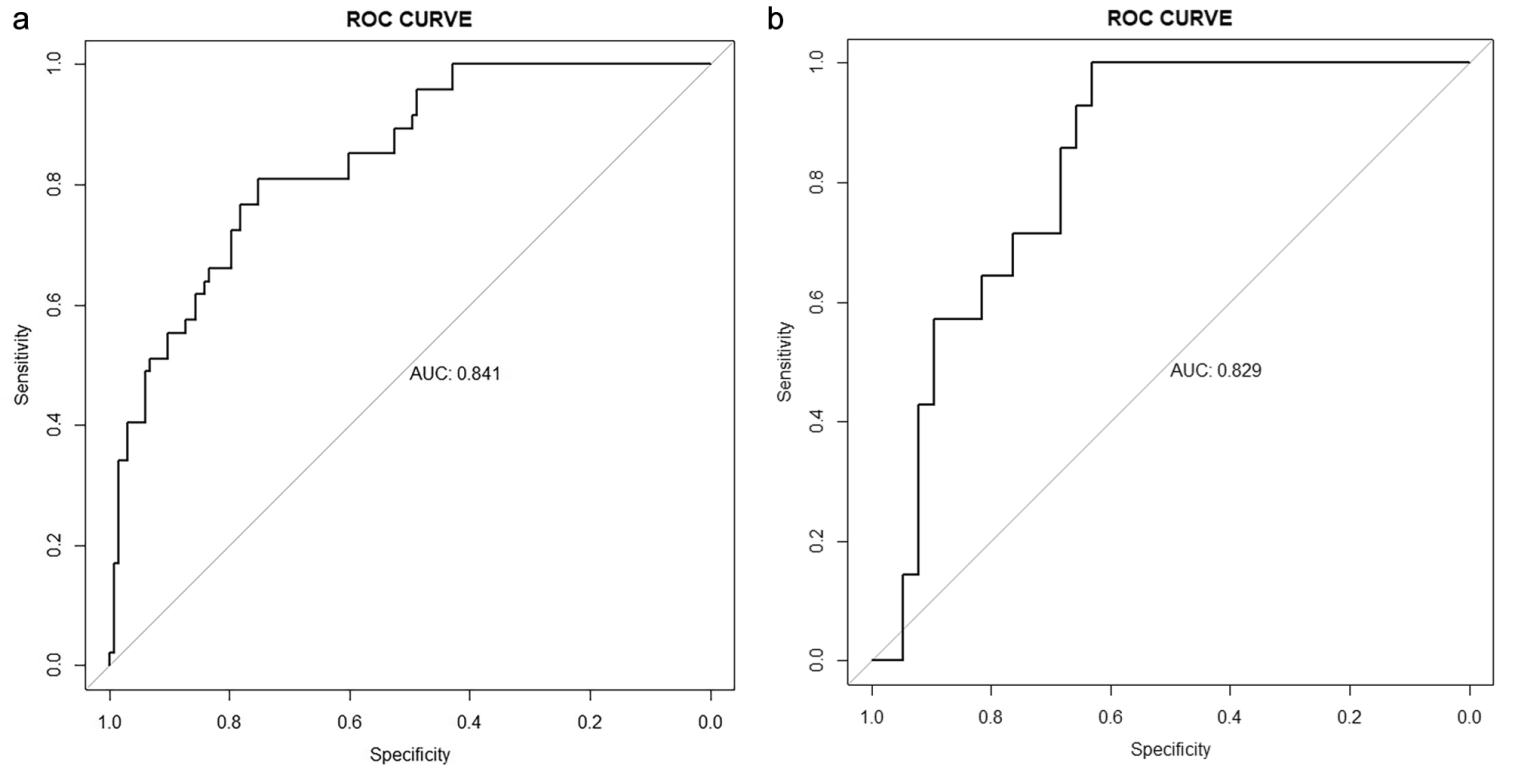
The ROC curves based on validation set for the diagnosis of PEW. The ROC curve was constructed to evaluate the diagnostic performance of final model. (**a**) ROC curve of the model in internal validation set. (AUC = 0.841, n = 161, p < 0.05) (**b**) ROC curve of the model in external validation set. (AUC = 0.829, n = 52, p < 0.05)

**Fig. 6:**
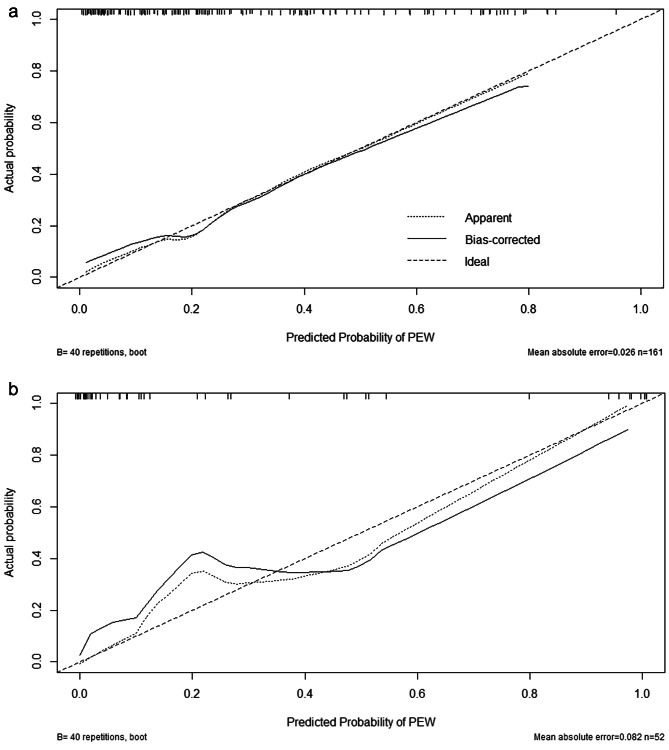
Calibration plot of final model by validation set. The graphs represent the relationship between observed and predicted PEW risk. The y-axis represents the actual PEW risk. The x-axis represents the predicted PEW risk. Dotted line is the performance of the model, of which a closer fit to the diagonal line represents a better prediction, while the solid line corrects for any bias in the model. Dashed line is the reference line. (**a**) Calibration curve of the model in internal validation set. (**b**) Calibration curve of the model in external validation set

ROC curves were built for internal and external validation set based on the final diagnostic model. The area under the curve (AUC) was 0.841 (95% CI: 0.806 ~ 0.880) in the internal validation set and 0.829 (95% CI: 0.719 ~ 0.939) in the external validation (Fig. 5[a-b]). Moreover, the calibration curve revealed good agreement between prediction by the nomogram and the actual observations in both internal and external validation set (Fig. 6[a-b]).

### Diagnostic test evaluation

In comparing the results of our final diagnostic model (we call it “BIA + PEW model”) with other previous diagnostic models (“PEW score model”, “modified PEW score model”, “SGA model”, “3-index model”, “BIA decision tree model”) against the ISRNM diagnostic criteria, a comparison table (Table 3) was developed. The cutoff values of these models were calculated by the principle of “Youden Index (YI) maximum” with our internal validation set. The detailed diagnostic criteria for other models are listed in Table supplementary 1, 4, 5, 6 and Figure supplementary 1 [17, 24–27]. The “BIA + PEW model” identified good sensitivity (73.2%) and specificity (78.3%). In general, Youden Index of “BIA + PEW model” is higher than that of other models. NRI of “BIA + PEW model” is 8%, 13%, 2%, 38%, 36% over “PEW score model”, “modified PEW score model”, “3-index model”, “SGA model”, “BIA decision tree model”, respectively. (all above p < 0.05). Other components in evaluating the validity of these diagnostic methods are listed in Table 3.

**Table 3:** Diagnostic test evaluation

Models	Cutoff	Result	Gold standard		C	Sen	Sp	PPV	NPV	YI	NRI
	value		Positive	Negative	statistics						
BIA + PEW	≥ 0.29	+	30	26	0.841	73.2%	78.3%	53.6%	89.5%	0.51	/
(risk 0 ~ 1)		−	11	94							
PEW score (score 0 ~ 4)	≤ 2	+	27	28	0.828	65.9%	76.7%	49.1%	85.2%	0.43	8%
		−	14	92							
Modified PEW score	≤ 2	+	28	37	0.809	68.3%	69.2%	43.1%	86.5%	0.38	13%
(score 0 ~ 4)		−	13	83							
3-index	≥ 0.24	+	31	32	0.822	75.6%	73.3%	49.2%	89.8%	0.49	2%
(risk 0 ~ 1)		−	10	88							
SGA	≥B	+	10	14	0.565	24.4%	88.3%	41.7%	77.4	0.13	38%
(grade A ~ C)		−	31	106							
BIA decision tree	≥ 2	+	11	14	0.573	26.8%	88.3%	44.0%	77.9	0.15	36%
(grade 1 ~ 3)		−	30	106							

Diagnostic test evaluation indicators include C statistics, sensitivity, specificity, positive predictive value, negative predictive value, Youden Index and net reclassification improvement. Cutoff value refers to that only when the corresponding risk, grade, and score are within the range, positive diagnosis of PEW can be established. NRI in this table refers to the higher reclassification ability of “BIA + PEW model” over other previous models

## Discussion

In this study, we proposed a BIA + PEW model for PEW diagnosis, which is suitable for Chinese maintenance dialysis patients. The model shows good discrimination and calibration in both internal and external validation, and has higher diagnostic accuracy than some existing diagnostic models. We find that BIA indicators can be used as good predictors of PEW, and the combination of BIA indexes (BCM, water ratio, VFA, phase angle) and single nutritional indicator from ISRNM (that is, cholesterol) has a high predictive value for PEW. These objective parameters included in the model are based on regular laboratory results, consequently cost-effective and easy to carry out.

Diagnosis of PEW is a challenging theme. Because there has been no single diagnostic marker or tool to perfectly determine whether a patient is PEW or not, clinical studies focusing on PEW inevitably require diagnostic definition of PEW by combining one or more of the nutrition-related surrogates to allocate patients into a binary variable pertaining to PEW. According to ISRNM, PEW diagnostic standard includes biochemical indicators, BMI, muscle mass, and diet. Optimally, each criterion should be documented on at least three occasions, preferably 2–4 weeks apart [1]. This diagnostic standard includes longitudinal data, such as changes in body weight and muscle mass over a period of time, which may require dynamic and multiple observations, causing inconvenience to the diagnosis of PEW. Thus, the practical application of the strict diagnostic standard in clinical practice is somewhat limited. Furthermore, the threshold for each of the four items is still controversial [2–4], and some indicators don’t fully reflect nutritional status. For example, a decrease in albumin may be a result of worsening liver function, while a decrease in muscle mass may be attributed to a natural process of aging [1]. Kovesdy et al. summarized the drawbacks of ISRNM critera [28]. In fact, each nutritional method should be adjusted depending on racial, ethnic and social backgrounds. However, there’s still a lack of PEW diagnostic standard targeted for large population of Chinese dialysis patients.

Several nutrition-related tests have been proposed to assess nutritional status. The 3-point scaled Subjective Global Assessment (SGA-3) [27] scores patients as A (well nourished), B (moderately malnourished) or C (severely malnourished) (Table supplementary 1). Although this test was validated in dialysis patients [9, 29], its semi-quantitative character and the fact that it does not adequately detect the degree of malnutrition [9] led to modifications like the 7-point scaled SGA (SGA-7) [9, 29] and the Malnutrition Inflammation Score (MIS) [30–32]. Other clinical nutritional scores or parameters that have been related to mortality in dialysis patients include the geriatric nutritional risk index (GNRI) [33–36], dialysis malnutrition score (DMS), and composite score of protein-energy nutritional status (cPENS) [37, 38]. It is currently unknown which test should be used to assess PEW most adequately [39]. In addition to above nutritional assessment means, Moreau-Gaudry et al. mentioned a “PEW score” tool, including 4 indicators of cross section (Table supplementary 4). The model has been proved to be able to predict the survival of dialysis patients [17]. As’habi et al. assessed PEW score with a high sensitivity of 100% but a low specificity of 28.6%, which may overestimate the risk of PEW [40]. Yamada et al. proposed modified PEW score, which was modified from the original simple PEW score by adjusting the cutoff values of those parameters suitable for Japanese patients receiving hemodialysis [24] (Table supplementary 5). Ruperto et al. proposed a model combining 3 nutrition-related indexes (serum albumin, percentage of mid-arm muscle circumference, standard body weight) to predict PEW risk, with a high AUC of 0.86 [26] (Table supplementary 6). However, the above tools solely use readily available clinical and biological values at bedside, without considering other components like appetite, dietary intake and physical examination.

In recent years, electrical bioimpedance has become the most useful, simple, and reproducible method for the study of body composition. According to the principle of Omron’s law, when the current passes through human tissues, it generates resistance and reactance. The resistance is related to the hydration state, while reactance is related to the capacitance. The composition of human body components can be derived by using the impedance value of current conduction in different tissues [41]. In a multi-frequency BIA machine, current frequency of 5 ~ 1000 kHz can be selected. At very low frequencies, virtually no conduction occurs because of high cell membrane capacitance, thus allowing for the quantification of ECW. At very high frequencies, total conduction through the cell membrane occurs, thus allowing for the quantification of TBW [42]. BIA is a practical method mainly used nowadays to assess dry weight, and it has been proven to be as accurate as the reference methods considered as the gold standard [43]. In this study, we find that there’s a certain correlation between BIA indexes and PEW. Water ratio is an independent risk factor, while BCM, VFA and phase angle are independent protective factors of PEW. Zhou et al. mentioned that increased volume load was an independent risk factor for PEW [44]. Dekker et al. also found that the higher the volume load was, the worse the nutritional status was, which is partially consistent with the results of this study [45]. Rymarz et al. found that the BCM level of hemodialysis patients was positively correlated with creatinine and handgrip strength, which are indicators of muscle mass, and negatively correlated with interleukin 6. By monitoring changes of BCM, the composition of muscle tissue can be observed at an early stage [12]. Valente et al. found that BCM was an independent factor for PEW, which excludes ECW, avoiding a possible masking of the nutritional status [46]. Ruperto et al. confirmed that PA < 4 ° is an independent risk factor for PEW [26], which is similar to our results. Bansal et al. demonstrated that phase angle was significantly associated with mortality in patients with CKD and hemodialysis [47]. By evaluating and observing the changes of the above indicators, it is helpful to identify PEW at early stages and take measures to reduce the incidence of PEW. Also, we find that compared with a single indicator from ISRNM to diagnose PEW, the combination of BIA indexes and single ISRNM indicator has a better predictive ability for PEW. This observation is acceptable because each marker provides only partial information on nutritional status. The combination of multiple surrogates enables us to assess nutritional status in a multifaceted way and offers a better prediction than a single surrogate. Currently, models have been developed for screening and diagnosing PEW in dialysis patients by using BIA. Wieskotten et al. proposed a decision tree model, which divided participants into adequate nutritional status, nutrition monitoring needed and insufficient nutritional status based on BIA measurement results [25] (Figure supplementary 1). Arias-Guillen et al. confirmed that the decision tree for nutritional status assessment was a practical tool for classifying patients, and by using this method, ‘insufficient nutritional status’ was an independent diagnostic factor of PEW. Combined with other nutritional assessment methods, this decision flowchart can provide additional value for selecting patients who need to focus on nutritional intervention in clinical practice [48].

Similarly, our BIA + PEW model also combined BIA indexes and ISRNM nutritional indicators. The model has an area under the curve of 0.843 and shows good discrimination and calibration in both internal and external validation. In the diagnostic test evaluation, we divided participants from the internal validation set into negative and positive groups using different PEW diagnostic methods. Compared to previous models, our BIA + PEW model has the highest C-index and NRI. This can be explained as follows. For SGA, its semi-quantitative character leads to difference of results from different observers. Only 27.5% of the patients with PEW were identified by SGA in our research, indicating the unreliability of SGA results. The results of PEW score and modified PEW score model are presented as 4 levels (severe waste, moderate waste, slight waste, normal nutritional status), the exact diagnostic bivariate thresholds for PEW of which have not been established. Therefore, we selected the optimal cutoff value as 2 by the principle of “Jordan Index maximum”, the score lower than which was diagnosed as PEW. In addition, the performance of 3-index model is slightly inferior to our model though it was established using the same logistic regression method as our BIA + PEW model. The decision tree model shows high specificity (88.3%) but low sensitivity (26.8%) in our research. These can be explained that these models originated from France, Japan, Spain and Germany, respectively, and there are slight differences in indicators from different races and populations, resulting in poor recognition of PEW in Chinese dialysis patients.

The present study has as main strengths the total number of patients studied, adequate internal and external validation. But some weaknesses and limitations of this study should be considered. On the one hand, although BIA was also shown to be a valid method for assessing body fluids in persons with varying hydration status in some study [49], most experts believe that it’s still not valid in subjects with altered hydration status [50–53]. Ho et al. evaluated the accuracy of BIA against multiple dilution (gold standard to detect TBW) to measure TBW in individuals pre- and post-dialysis, which showed no statistically difference between them in terms of TBW average and reasonably better agreement between the two methods at post-dialysis moments than at pre-dialysis moments [54]. So in our study, BIA index measurement time point is limited to 15 min after the end of dialysis, which to the greatest extent limits the imprecision caused by the unstable volume load, though it does not rule out the measurement error caused by insufficient or excessive dialysis completely. Thus, bioelectrical impedance vector analysis (BIVA), proposed by Piccoli et al. in 1994 [53], which is reported to be an alternative method that has been validated and used for hydration status and body composition assessment in different populations, may help to further expand the validity of this study. Chamney model, proposed by Chamney et al. has been used in some BIA devices, which can distinguish muscle mass from the fluid overload and differentiate excess fluid from normally hydrated tissue, thus providing meaningful estimates of nutrition assessment for dialysis patients [22]. Moreover, our data only includes baseline levels of nutritional markers instead of repeated measures. Furthermore, as an observational study from single center, it is difficult to account unmeasured or residual confounding factors, which can lead to bias. However, though cross-sectional nature of the study may limit accuracy partially, the proposed diagnostic method can diagnose PEW quickly, conveniently, and economically, which fits the purpose of our research well and may have great value in clinical application. What’s more, though the model shows high AUC of 0.829 in external validation, the sample size of external validation is too small, resulting in wide confidence interval and calibration with slightly higher deviation. It is unclear whether our BIA + PEW model is a good predictor of clinical outcomes such as death, cardiovascular disease events, bone fracture, or hospitalization. Therefore, further studies of larger samples are necessary to determine the usefulness and validity of the model developed in our study.

In conclusion, it is hard to assess PEW in maintenance dialysis patients in daily clinical practice. Based on the recommendations of ISRNM, we suggest a new combination of parameters, which are readily available and strongly associated with other nutritional parameters. A single index of ISRNM combined with BIA indexes can also well diagnose PEW and evaluate its risk when it is impossible to obtain all the PEW diagnostic criteria.

### Electronic supplementary material

Below is the link to the electronic supplementary material.

Supplementary Material 1

## Data Availability

The dataset supporting the conclusions of this article is included within the article.

## List of abbreviations

AMC: arm muscle circumference
AUC: area under the curve
BCM: body cell mass
BFP: body fat percentage
BIA: bioelectrical impedance analysis
BIVA: bioelectrical impedance vector analysis
BMI: body mass index
BSA: body surface area
CKD: chronic kidney disease
cPENS: composite score of protein-energy nutritional status
DMS: dialysis malnutrition score
ECW: extracellular water
ESRD: end-stage renal disease
FFM: fat free mass
FTI: fat tissue index
GNRI: geriatric nutritional risk index
IQRs: interquartile ranges
ISRNM: International Society of Renal Nutrition and Metabolism
Kt/V: urea clearance index
LTI: lean tissue index
MIS: malnutrition-inflammation score
nPCR: normalized protein catabolic rate
nPNA: normalized protein nitrogen appearance
NPV: negative predictive value
NRI: net reclassification improvement
OH: overhydration
OR: odds ratio
PEW: protein-energy wasting
PPV: positive predictive value
ROC: receiver operating characteristic
SBW: standard body weight
SDs: standard deviations
SGA: subjective global assessment
SGA-3: 3-point scaled subjective global assessment
SGA-7: 7-point scaled subjective global assessment
SLM: soft lean mass
SMM: skeletal muscle mass
TBW: total body water
TST: triceps skinfold thickness
VFA: visceral fat area
YI: Youden Index
