# Supplementary material for: Study on the correlation between bioelectrical impedance analysis index and protein energy consumption in maintenance dialysis patients
Source: Nutr J. 2023 Nov 9;22:56. doi: 10.1186/s12937-023-00890-5 (PMC10633946; doi:10.1186/s12937-023-00890-5)
Supplement: Supplementary file 1 — Supplementary Material 1 [file 12937_2023_890_MOESM1_ESM.docx]

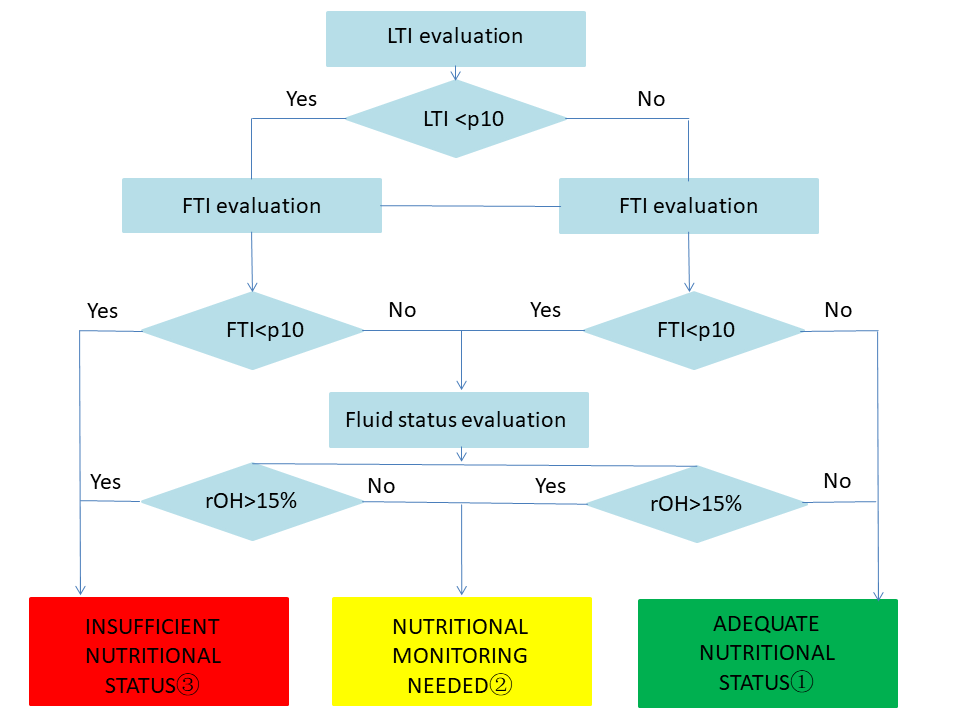


**Figure supplementary 1.Decision tree for the assessment of nutritional status.** This graph shows a method to stratify nutritional status by evaluating LTI, FTI and rOH. By using this method, we divided validation set into 3 nutritional status. After determining optimal cutoff value, we define insufficient nutritional status group and nutritional monitoring needed group as PEW positive groups. p10, tenth percentile of population; LTI, lean tissue index; FTI, fat tissue index; rOH: relative overhydration.

**Table supplementary 1. Main criteria for subjective global assessment (SGA)**

| SGA A | <5% weight loss |
| --- | --- |
| SGA B | 5-10% weight loss |
|  | Minor loss of subcutaneous fat mass |
|  | Minor loss of muscle mass |
| SGA C | >10% weight loss |
|  | Major loss of subcutaneous fat mass |
|  | Major loss of muscle mass |
|  | Oedema |

SGA=B and C is defined as malnutrition in this study.

**Table supplementary 2. Characteristics of training set and validation set**

| Characteristics | Overall(n=609) | | P value |
| --- | --- | --- | --- |
|  | Training set(n=448) | Validation set(n=161) |  |
| PEW |  |  |  |
| Yes | 109(24.3%) | 44(27.3%) | 0.452 |
| No | 339(75.7%) | 117(72.7%) |  |
| Sex |  |  |  |
| Male | 223(49.8%) | 89(55.3%) | 0.231 |
| Female | 225(50.2%) | 72(44.7%) |  |
| Age, y | 54.08±13.67 | 53.16±13.29 | 0.458 |
| Dialysis modality |  |  |  |
| Peritoneal dialysis | 252(56.3%) | 86(53.4%) | 0.535 |
| Hemodialysis | 196(43.7%) | 75(46.6%) |  |
| Dialysis duration, m | 36(19.0,63.8) | 39(18.0,66.5) | 0.971 |
| SGA |  |  |  |
| Yes | 68(15.2%) | 24(14.9%) | 0.934 |
| No | 380(84.8%) | 137(85.1%) |  |
| Albumin, g/L | 39.99±3.81 | 40.22±3.81 | 0.517 |
| Prealbumin, g/L | 39.23±9.78 | 39.16±9.45 | 0.204 |
| Cholesterol, mmol/L | 4.06±1.01 | 4.03±1.04 | 0.690 |
| Triglyceride, mmol/L | 1.73(1.19,2.50) | 1.64(1.18,2.59) | 0.960 |
| nPCR, g/(kg·d) | 0.99±0.32 | 0.99±0.30 | 0.921 |
| C-reactive protein, mg/L | 1.62(0.53,4.72) | 1.57(0.57,5.34) | 0.847 |
| Serum creatinine, μmol/L | 921.59±278.39 | 908.88±292.44 | 0.624 |
| Kt/V | 1.86±0.51 | 1.79±0.48 | 0.171 |
| Hemoglobin, mg/L | 105.50±13.85 | 107.02±15.45 | 0.247 |
| Urea nitrogen, mmol/L | 23.31±6.20 | 22.27±6.09 | 0.069 |
| Calcium, mmol/L | 2.26±0.23 | 2.25±0.19 | 0.643 |
| Phosphorus, mmol/L | 1.78±0.45 | 1.74±0.56 | 0.423 |
| Parathyroid hormone, pg/mL | 284.7(144.0,477.0) | 318.0(167.0,478.0) | 0.574 |
| Serum iron, μmol/L | 11.8(9.0,15.1) | 11.6(8.7,15.2) | 0.928 |
| Ferritin, pg/mL | 155.3(70.4,324.1) | 127.0(55.3,316.3) | 0.095 |
| Arm circumference, cm | 26.95±3.74 | 26.71±3.54 | 0.465 |
| AMC, cm | 21.93±3.02 | 21.69±2.40 | 0.364 |
| TST, mm | 15.76(11.78,19.43) | 15.92(11.62,19.11) | 0.856 |
| BMI, kg/m^2^ | 21.83±3.42 | 21.63±3.23 | 0.506 |
| FFM, kg | 40.8(30.2,51.1) | 41.1(31.8,50.2) | 0.758 |
| BFP, % | 28.2(18.0,48.1) | 27.8(19.2,44.4) | 0.604 |
| FAT, kg | 17.56(10.92,30.44) | 17.33(9.87,27.10) | 0.527 |
| BCM, kg | 27.48±7.37 | 27.65±6.59 | 0.805 |
| Water ratio(ECW/TBW),% | 39.08±3.17 | 38.82±3.09 | 0.379 |
| ECW, kg | 13.01±5.08 | 12.66±4.40 | 0.439 |
| SLM, kg | 40.58±10.64 | 40.77±975 | 0.841 |
| VFA, cm^2^ | 53.3(31.8,76.3) | 49.1(31.1,73.0) | 0.201 |
| Impedace, Ω | 1010.86±199.26 | 1012.50±186.14 | 0.927 |
| Phase angle, ° | 6.15±2.22 | 6.03±1.62 | 0.470 |

## SGA-Yes: malnutrition evaluated through subjective global assessment. SGA-No: good nutritional status evaluated through subjective global assessment. PEW, protein-energy wasting; nPCR, normalized protein catabolic rate; AMC, arm muscle circumference; TST, triceps skinfold thickness; BMI, body mass index; FFM, fat free mass; BFP, body fat percentage; BCM, body cell mass; ECW, extracellular water; SLM, soft lean mass; VFA, visceral fat area.*: p<0.05; **: p<0.01.

**Table supplementary 3. Result of collinearity diagnosis in BIA+PEW model**

| Indicators | Variance inflation factor |
| --- | --- |
| Water ratio | 1.155 |
| VFA | 1.312 |
| BCM | 1.219 |
| Phase angle | 1.090 |
| Cholesterol | 1.035 |

BCM, body cell mass; VFA, visceral fat area.

**Table supplementary 4. Definition of the PEW score**

| Indexes | Threshold value |
| --- | --- |
| Albumin (g/dL) | <=3.8 |
| BMI(kg/m2) | <=23 |
| Scr/BSA(umol/L/m2) | <=380 |
| nPNA(g/kg/d) | <=0.8 |

## PEW score is defined as the grading of 1 selected item in each of the 4 categories of the wasting syndrome: (1) serum albumin, (2) BMI, (3) pre-dialysis serum creatinine normalized by body surface area, (4)normalized protein nitrogen appearance. The threshold values were shown in table supplementary 4. After selecting the optimal cutoff value, total score<=2 is defined as PEW in this study. BMI, body mass index; BSA, body surface area; nPNA, normalized protein nitrogen appearance.

**Table supplementary 5. Definition of modified PEW score**

| Indexes | Threshold value |
| --- | --- |
| Albumin (g/dL) | <=3.8 |
| BMI(kg/m2) | <=18.5 |
| Scr/BSA(umol/L/m2) | <=500 |
| nPNA(g/kg/d) | <=0.8 |

## Modified PEW score is defined as the grading of 1 selected item in each of the 4 categories of the wasting syndrome: (1) serum albumin, (2) BMI, (3) pre-dialysis serum creatinine normalized by body surface area, (4)normalized protein nitrogen appearance. The threshold values were shown in table supplementary 5. After selecting the optimal cutoff value, total score<=2 is defined as PEW in this study. BMI, body mass index; BSA, body surface area; nPNA, normalized protein nitrogen appearance.

**Table supplementary 6. Definition of 3-index model**

| Indexes | Notes |
| --- | --- |
| Albumin (g/dL) | / |
| Percentage of AMC | regarding normative reference Spanish population of same sex, height and age range |
| percentage of SBW | SBW (%) = (actual weight/SBW) /100, |

3 indexes from the table are included to construct a model by logistic regression. The actual percentage of AMC according to our internal validation set is calculated regarding normative reference Chinese population of same sex, height and age range. After selecting the optimal cutoff value, PEW risk≥0.24 is defined as PEW in this study. AMC, arm muscle circumference; SBW, standard body weight.
